# *De novo* transcriptome assembly of *Zanthoxylum bungeanum* using Illumina sequencing for evolutionary analysis and simple sequence repeat marker development

**DOI:** 10.1038/s41598-017-15911-7

**Published:** 2017-12-01

**Authors:** Shijing Feng, Lili Zhao, Zhenshan Liu, Yulin Liu, Tuxi Yang, Anzhi Wei

**Affiliations:** 1 College of Forestry, Northwest A&F University, Yangling, Shaanxi 712100 China; 2 College of Life Science, Northwest A&F University, Yangling, Shaanxi 712100 China

## Abstract

*Zanthoxylum*, an ancient economic crop in Asia, has a satisfying aromatic taste and immense medicinal values. A lack of genomic information and genetic markers has limited the evolutionary analysis and genetic improvement of *Zanthoxylum* species and their close relatives. To better understand the evolution, domestication, and divergence of *Zanthoxylum*, we present a *de novo* transcriptome analysis of an elite cultivar of *Z. bungeanum* using Illumina sequencing; we then developed simple sequence repeat markers for identification of *Zanthoxylum*. In total, we predicted 45,057 unigenes and 22,212 protein coding sequences, approximately 90% of which showed significant similarities to known proteins in databases. Phylogenetic analysis indicated that *Zanthoxylum* is relatively recent and estimated to have diverged from *Citrus ca*. 36.5–37.7 million years ago. We also detected a whole-genome duplication event in *Zanthoxylum* that occurred 14 million years ago. We found no protein coding sequences that were significantly under positive selection by *Ka*/*Ks*. Simple sequence repeat analysis divided 31 *Zanthoxylum* cultivars and landraces into three major groups. This *Zanthoxylum* reference transcriptome provides crucial information for the evolutionary study of the *Zanthoxylum* genus and the Rutaceae family, and facilitates the establishment of more effective *Zanthoxylum* breeding programs.

## Introduction

*Zanthoxylum* L. of the Rutaceae family is a species-rich genus, widely distributed in tropical and subtropical areas. The *Zanthoxylum* genus consists of >250 species of deciduous and evergreen trees, shrubs, and dunga-runga, with 39 species and 14 varieties being found in China^1,2^. *Zanthoxylum* has a long history of cultivation and domestication for both economic and chemical value in Asia. This genus is a promising source of secondary metabolites including alkylamides^3^, alkaloids^4^, and flavonoids^5^. Species within this genus can be used as spices and in traditional medicine.

In contrast to studies of phytochemistry and biological activity^6–8^, historical and evolutionary analysis of *Zanthoxylum* has received little attention. *Zanthoxylum* has a rich genetic patrimony in China and is characterized by an abundance of cultivars, some of which have a unique botanical feature of nucellar embryony^9^. This process of apomixis has hindered genetic study and breeding improvement of *Zanthoxylum*, because the seedlings are essentially clones of the maternal parent. Moreover, empirical observations indicate that the classification of the commercial classes of *Zanthoxylum* cultivars is often confusing because of homonyms, synonyms, and scarce or incorrect historical records.

The lack of genetic resources and genomic information has hampered the improvement of *Zanthoxylum* to enhance yield and resistance to biotic and abiotic stresses. Additionally, inappropriate selection of cultivars in agricultural production of *Zanthoxylum* can cause economic loss. Therefore, reliable and distinguishable genetic markers are a requirement for genetic and breeding studies of *Zanthoxylum*, enabling the development of improved genotypes or varieties with enhanced trait values.

Complete information on transcriptomes can easily be generated via high-throughput mRNA sequencing technology, which is a powerful and cost-effective tool for gene expression profiling in non-model organisms without a reference genome^10^. Illumina transcriptome *de novo* sequencing and assembly have been successfully used for Rutaceae plants, with recent research focusing on *Citrus* ^11–14^ and *Pilocarpus* species^15^. To the best of our knowledge, no studies have reported transcriptome sequencing of *Zanthoxylum*.

In this study, we present the first transcriptome of a *Zanthoxylum* species, for *Z. bungeanum*, which is native to China. *Z. bungeanum* has become the leading commercially cultivated species in this country because of its fast growth and adaptability to adverse soil and climatic conditions^16^. We aimed to: i) characterize the *Z*. *bungeanum* transcriptome for gene content; ii) describe the evolutionary features of *Zanthoxylum*; and iii) develop simple sequence repeat (SSR) markers for *Zanthoxylum* species. The transcriptome analysis presented in this study yields new insights into the evolutionary origin of *Zanthoxylum* and provides a rich resource of genetic information for breeding and genetic improvement.

## Results

### Sequencing and *de novo* assembly of Illumina paired-end reads

In this study, we generated sequences for the transcriptome of an elite *Z*. *bungeanum* cultivar, ‘Fengxiandahongpao’. Approximately 56.2 million high-quality reads were generated, with a GC content of 43.38%. The combined sequences of high-quality reads were assembled into 65,337 individual transcripts and 45,057 unigenes, reaching a total length of 41.0 Mb and 27.5 Mb, respectively. The average length of the assembled transcripts was 627 bp, with an N50 of 874 bp. The length of the assembled unigenes ranged from 201 to 6,750 bp, with an average of 610 bp and an N50 of 846 bp (Table 1).

**Table 1:** Summary for the transcriptome of *Z*. *bungeanum* cultivar.

Total number of reads	56,242,196
Total number of transcripts	65,337
Total number of unigenes	45,057
Average transcript length (bp)	627
Average unigenes length (bp)	610
N50 of transcript (bp)	874
N50 of unigenes (bp)	846
Longest unigenes (bp)	6,750
Shortest unigenes (bp)	201
GC content 43.38%	

**Figure 1:**
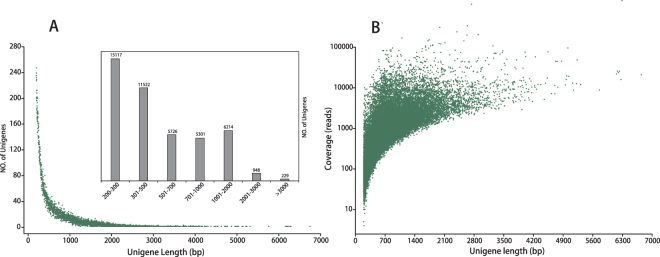
Distribution of unigene length and coverage depth in the transcriptome assembly of *Z. bungeanum*. (**A**) Length distribution of the assembled unigenes. (**B**) Density scatter-plot showing the relationship between unigene length and coverage.

Of all the unigenes, those from 201 to 300 bp accounted for 33.55% (15,117). The unigenes ranging from 301 to 500 bp accounted for 25.57% (11,522). There were 5,726 unigenes (12.71%) with lengths ranging from 501 to 700 bp, and 5,301 unigenes (11.77%) with lengths ranging from 701 to 1,000 bp. Only 2.61% of the unigenes (1177) were longer than 2,000 bp (Fig. 1).

### Sequence annotation

For validation and annotation of the sequence assembly contigs and unique singletons, all unigenes were searched against six public protein databases, including the NCBI non-redundant protein (Nr) database, protein family (Pfam), Swiss-Prot, the Kyoto Encyclopedia of Genes and Genomes pathway (KEGG) database, the EuKaryotic Orthologous Groups (KOG) database, and the Gene Ontology (GO) database. In total, 40,223 of 45,057 unigenes (89.27%) provided significant BLAST results, with 40,214 (89.25%) showing significant similarity to known proteins in the Nr database, 28,644 (63.57%) to proteins in the Swiss-Prot, and 18,128 (40.23%) to proteins in the Pfam. Only 1,715 unigenes were annotated in the six databases (Supplement Figure S1).

To further analyze the BLAST results, *E*-value and similarity distributions were calculated using the Nr database. The *E*-value distribution of the top hits revealed that 78.77% of the mapped sequences showed significant homology (E-value < 10^−30^), and 21.22% of the homologous sequences had *E*-values in the range 10^−6^ to 10^−30^ (Supplement Figure S2A). Additionally, 91.18% and 76.17% of the sequences were found to have similarities >70% and 80%, respectively (Supplement Figure S2B). These results reflect the high identities of the mapped sequences, suggesting a good assembly quality.

The species distribution of the top BLASTx hits against the Nr database showed that *Z*. *bungeanum* genes had the greatest number of matches with genes of orange (*Citrus*), cacao (*Theobroma*), and cotton (*Gossypium*). Among these, *C. sinensis* ranked first, with 13.3% top BLASTx hits, followed by *T. cacao* (7.1%), *C. clementine* (6.2%), *G. hirsutum* (4.7%), and *G. raimondii* (4.6%) (Supplement Figure S2C).

### Functional classification by GO analysis

**Figure 2:**
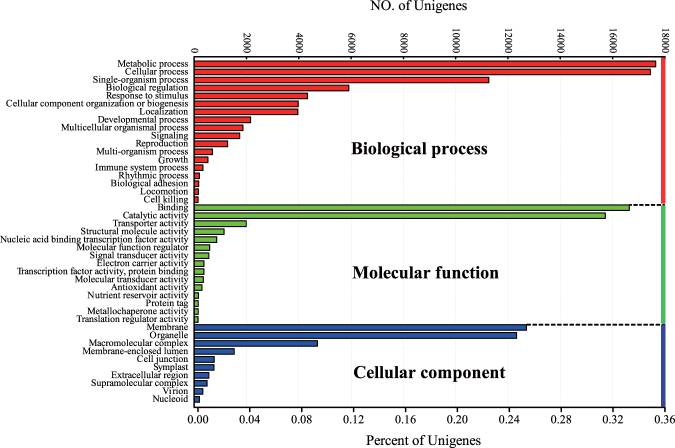
Gene ontology classification of assembled unigenes of *Z. bungeanum*. Results are summarized in three main categories: biological process, cellular component, and molecular function. The bottom x-axis indicates the percentage of a specific category of genes in that main category. The top x-axis indicates the actual number of genes in a category.

In total, 32,715 unigenes (72.61%) with BLAST matches to known proteins were assigned to GO classes with 132,647 functional terms (Fig. 2). The annotated unigenes that belonged to the biological process, cellular component, and molecular function clusters were categorized into 43 functional groups. Molecular function (47,706, 35.96%) and biological process (46,861, 35.33%) represented the largest number of unigenes, followed by cellular component (38,080, 28.71%).

Under the classification molecular function (in total 47,706), binding (16,797, 35.21%) and catalytic activity (15,869, 33.26%) were the largest and second largest categories, respectively. Other categories, such as transporter activity, structural molecule activity, nucleic acid binding transcription factor activity, and molecular transducer activity, contained 15,040 unigenes (31.53%) in total. Under the category biological process (in total 46,861), the most frequent subcategories were metabolic process (17,831, 38.05%) and cellular process (17,619, 37.60%), followed by single-organism process (11,325, 24.17%), biological regulation (5,869, 12.52%), and response to stimulus (4,265, 9.10%). Three categories, membrane, organelle, and macromolecular complex, represented approximately 38.75% of cellular component classifications (Fig. 2).

### Functional classification by KOG analysis

**Figure 3:**
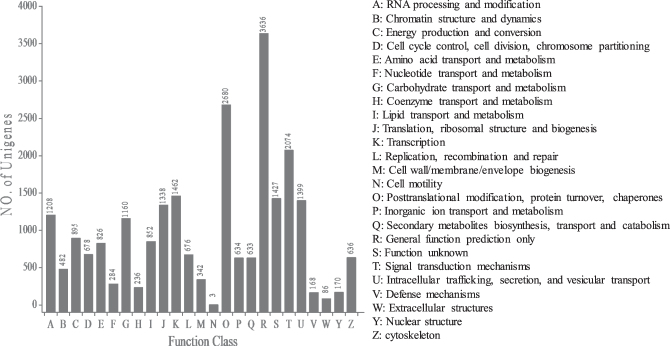
Histogram presentation of KOG classification of *Z. bungeanum* unigenes. Out of 48,300 Nr database hits, 3,112 sequences have a KOG classification among the 25 categories.

To further evaluate the completeness of our transcriptome library, all unigenes were searched against the KOG database for functional prediction and classification. In total, 31,657 of 45,057 unigenes (70.26%), showing significant homology to entries in the KOG database, were functionally classified into 25 molecular families (Fig. 3). Among these KOG categories, the largest group was “General function prediction only” containing 3,636 unigenes (15.16%), followed by “Posttranslational modification, protein turnover, chaperones” (11.17%), “Signal transduction mechanisms” (8.65%), “Transcription” (6.10%), “Function unknown” (5.95%), and “Intracellular trafficking, secretion, and vesicular transport” (5.83%). Only three unigenes were assigned to “Cell motility” (0.01%).

### Functional classification by KEGG pathways

**Figure 4:**
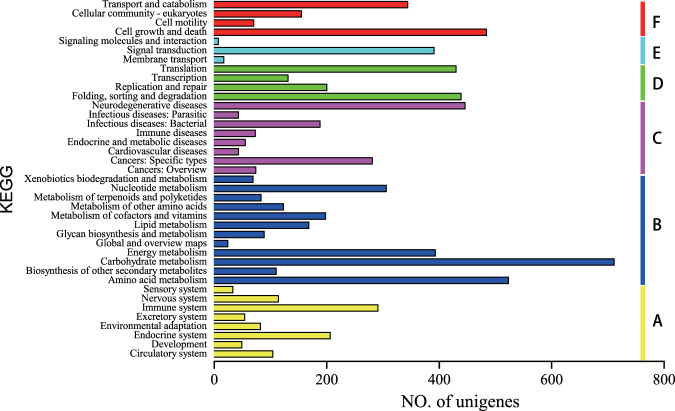
Pathway assignment based on KEGG pathways of *Z. bungeanum* unigenes. (**A**) Classification based on organismal systems categories. (**B**) Classification based on metabolism categories. (**C**) Classification based on human diseases categories. (**D**) Classification based on genetic information processing categories. (**E**) Classification based on environmental information processing categories. (**F**) Classification based on cellular process categories.

To further analyze the transcriptome of *Z*. *bungeanum*, all the unigenes were analyzed via the KEGG pathway database, where a total of 3,093 unigenes had significant matches and were assigned to six main categories in 229 pathways (Fig. 4). Among these six main categories, metabolism was the largest, containing 2,797 unigenes (90.43%), followed by genetic information processing (1,200, 38.80%), human diseases (1,076, 34.79%), cellular processes (1,047, 33.85%), organismal systems (886, 28.65%), and environmental information processing (415, 13.42%).

### Evolutionary pattern of protein coding sequences

**Figure 5:**
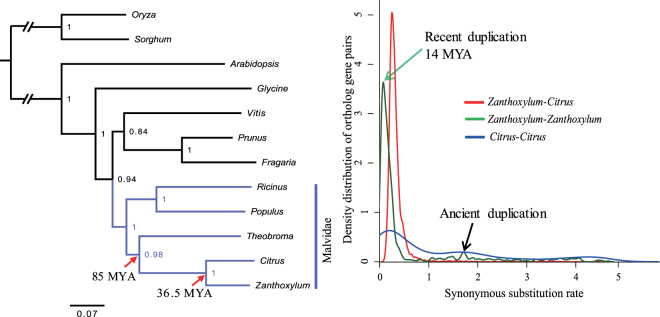
Evolutionary analysis of *Z*. *bungeanum*. (**A**) *Zanthoxylum* phylogeny on the basis of 34 single-copy nuclear genes shared by *Zanthoxylum* and other plant species. Rice (*Oryza sativa*) and sorghum (*Sorghum bicolor*) were used as out-groups. MYA, million years ago. (**B**) Distribution of synonymous substitution rates (*K*s) for homologous gene groups in protein coding sequences (CDS) of *Zanthoxylum* and *Citrus*.

The protein coding sequences (CDS) extracted from unigenes also provided comprehensive information for phylogenetic analysis. A phylogenetic analysis was conducted based on the 34 single-copy nuclear genes (Supplement Dataset S1) shared in *Zanthoxylum*, sweet orange (*Citrus sinensis*), poplar (*Populus euphratica*), grape (*Vitis aestivalis*), strawberry (*Fragaria* × *ananassa*), soybean (*Glycine max*), *Arabidopsis*, cacao (*Theobroma cacao*), peach (*Prunus persica*), and castor bean (*Ricinus communis*). *Zanthoxylum* was found to be phylogenetically closest to sweet orange, cacao, poplar, and castor bean (Fig. 5A). For comparison, a phylogenetic tree was derived from 68 chloroplast genes (Dataset S2) in the same species, which showed that *Zanthoxylum* was phylogenetically closest to sweet orange, cacao, *Arabidopsis*, and grape (Supplement Figure S3). This indicated a significantly different evolutionary pattern between or within nuclear and plastid genomes in some plant species (*e.g*., grape and *Arabidopsis*). Assuming orange diverged from cacao 85 million years ago (MYA)^17^, we confidently estimated a divergence time for *Zanthoxylum* and *Citrus* of 36.5 and 37.7 MYA from the nuclear and chloroplast trees, respectively (Fig. 5A and S3).

Meanwhile, an age distribution was calculated by comparing the synonymous substitution values (*Ks*) of 2,750 orthologous gene pairs between the *Zanthoxylum* and *Citrus* genomes, 794 paralogous gene pairs in the *Zanthoxylum* transcriptome, and 1,054 paralogous gene pairs in *Citrus* genomes. A *Zanthoxylum* whole-genome duplication (WGD) event occurred 14 MYA (*Ks* peaks at 0.074), more recently than the divergence of *Zanthoxylum* and *Citrus*. Interestingly, we detected three peaks in the *Ks* distribution in orange, suggesting that at least three ancient WGD events occurred in orange (Fig. 5B). A second large *Ks* peak at a synonymous substitution rate of about 1.7 was observed in both *Zanthoxylum* and *Citrus*, indicating that an ancient WGD event occurred in their common ancestor (Fig. 5B and Supplement Figure S4). We also inferred the direction and magnitude of natural selection acting on protein coding genes in *Zanthoxylum* by calculating the *Ka*/*Ks* through referring to *Citrus* orthologous genes. Only four genes with *Ka*/*Ks* > 1 but non-significant were observed, implying that most *Zanthoxylum* genes experienced purifying or stabilizing selection (Supplement Figure S5).

To better understand *Zanthoxylum*-specific biology, we identified *Zanthoxylum*-specific genes by comparative analyses of the *Zanthoxylum* protein CDS with 12 other plant genomes and rescreened them in the Nr protein database to exclude false-positive genes caused by incomplete genome annotation. In total, 22,212 CDS in the *Zanthoxylum* transcriptome were clustered into 8154 gene families, with 173 genes being specific to *Zanthoxylum* (Supplement Figure S6). One hundred of the *Zanthoxylum*-specific genes had unknown function. Of the 73 annotated genes, overrepresented molecular functions were oxidation-reduction, regulation of transcription, and translation processes. Exploring the function of these genes will contribute significantly to understanding of important *Zanthoxylum*-specific biological processes, such as the synthesis of numb-taste components.

### Identification of single nucleotide polymorphisms

From the *Z. bungeanum* transcriptome sequencing data, we identified 170,000 single nucleotide polymorphisms (SNPs) in 37,658 unigenes. The proportions of transversions were: 10.48% A/C; 12.44% A/T; 7.04% C/G and 10.33% G/T. The proportions of transitions were: 29.61% A/G and 30.10% C/T. The percentage of transitions (59.71%) was 1.48-times higher than that of transversions (40.29%) in the *Z. bungeanum* transcriptome. Further analysis of the SNPs revealed that approximately 72% of them were distributed in CDS, and 20,461 CDS contained at least one SNP, leading to a rough estimate of polymorphism density of 6.16 SNPs per kb in sequences containing CDS. The large number of SNPs located in sequences containing CDS is possibly beneficial for future development of important economic and agronomic traits. These SNPs could be useful for molecular breeding programs and marker-assisted selection practices, as well as for understanding phenotypic differences between *Zanthoxylum* species.

### Identification of simple sequence repeats from *Z*. *bungeanum* transcriptome

**Figure 6:**
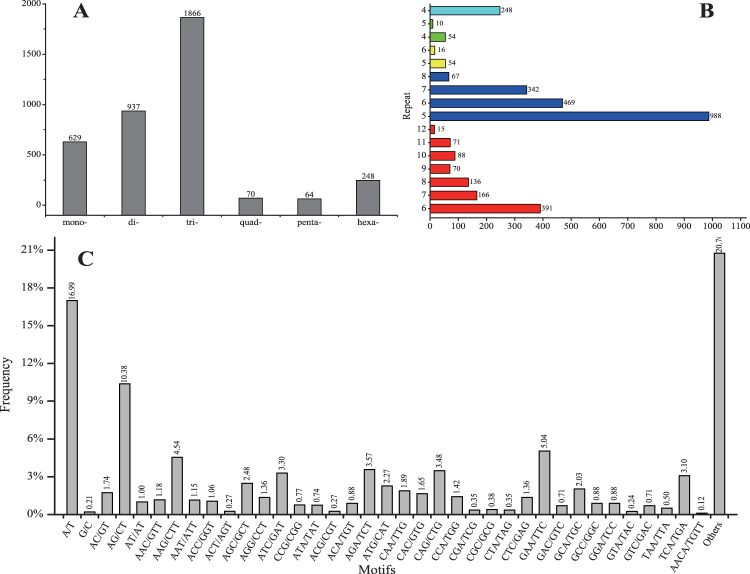
Results of microsatellite SSR analyses in *Z. bungeanum*. (**A**) Distribution of the total number of SSRs in different classes of repeat type. Di-, tri-, tetra-, penta-, and hexanucleotide repeats were analyzed. (**B**) Distribution of the number of SSR repeats. (**C**) Frequency distribution of SSRs based on motif sequence type.

A total of 3,814 potential simple sequence repeats (SSRs) were identified across 45,057 unigenes from the transcriptome of *Z*. *bungeanum*. Of these, 488 unigenes (12.79%) contained one or more SSR sequences, 282 unigenes (7.39%) contained at least two separate SSR sequences, and 206 unigenes (5.40%) contained compound SSRs of different motifs (Fig. 6A). The number of SSRs per unigene ranged from 1 to 5. Trinucleotide repeat motifs were the most frequent type (1866, 48.93%) with 5–8 repeats, followed by 937 (24.57%) di-type motifs with 6–12 repeats, 629 (16.49%) mono-type motifs, and 248 (6.50%) hexa-type motifs with 4 repeats. Fewer quad-type motifs (70, 1.84%) and penta-type motifs (64, 1.68%) were observed, with 5–6 and 4–5 repeats, respectively (Fig. 6B).

Except for compound SSRs, A/T was the most abundant motif in the two possible types of mono-nucleotide repeat, contributing 16.31% of the total SSRs. The C/G motif was less abundant than A/T, accounting for 0.21% of the total SSRs. The dominant di-nucleotide repeat motif in SSRs was AG/CT accounting for 10.38% of the total SSRs. The AC/GT motif was slightly more abundant than the AT/AT motif, which constituted 1.74% and 1.00% of the total SSRs, respectively. Among the trinucleotide repeat motifs, the two most frequent repeats were GAA/TTC (5.04% of the total SSRs) and AAG/CTT (4.54%), followed by ATC/GAT (3.30%), AGA/TCT (3.57%), and CAG/CTG (3.48%). Other motifs among quad-, penta-, and hexa- nucleotide repeats constituted 20.88% of the total SSRs (Fig. 6C).

### Development and validation of SSR markers

Out of 3,814 SSRs, a total of 1,000 primer pairs were successfully designed; 100 primer pairs were selected at random and tested to amplify the genomic DNA of *Z. bungeanum* (20 cultivars and two landraces) and *Z. armatum* (five cultivars and four landraces). Sixty primer pairs resulted in successful PCR amplification, 25 of which produced fragments of the expected size. Taking into account the specific amplification and polymorphic loci, we selected 16 primer pairs that presented clear and polymorphic loci to help evaluate polymorphism levels in the two *Zanthoxylum* species. After further testing, we selected 11 primer pairs for subsequent polymorphic analysis (Table 2). Compared with *Z. armatum* cultivars, *Z. bungeanum* cultivars possessed a relatively high number of alleles (*N*_*a*_ = 4.636), effective number of alleles (*N*_*e*_ = 3.088), observed and expected heterozygosity (*H*_*o*_ = 0.749 and *H*_*e*_ = 0.655), and Shannon index (*I = *1.225). Additionally, the fixation index (*F*_*IS*_) of *Z. bungeanum* and *Z. armatum* varied from −0.485 to 0.367 and −0.385 to 0.600, with an average of −0.183 and 0.027, respectively (Supplement Table S2).

**Table 2:** Characteristics of primers designed for analyzing genetic diversity of *Zanthoxylum* cultivars.

Code	Primer pair	Length (bp)	Melting temperature (°C)	Product size (bp)	GC content (%)
ZB-2	F: AACAAATGGTGCTGATGACA	20	54	177	45.2
	R: ATTACTGCTCCTCGAAACTGG	21			
ZB-4	F: TCAACAGATATGGCTCGAGT	20	54	206	43.2
	R: TTTGAAACCCCGAACATGG	19			
ZB-11	F: AGGCTTTCAATGAGTTCAAGG	21	56	206	43.7
	R: CTCTTCTGGATTTTCCTCGT	20			
ZB-28	F: ACAACAGACTGCTAAGCCAA	20	54	139	39.6
	R: TCCTTTTGCCTTTCAACCC	19			
ZB-30	F: ACACTGATATACCGGCACCT	20	58	201	49.8
	R: ATGCCTCGTTTGGATCGCTT	20			
ZB-32	F: ATTCCGAAATCCCGCCCAA	19	56	180	47.2
	R: TTTCCGACAGCAACGGCTT	19			
ZB-43	F: CAGTCTCATTAAACGCTCC	19	54	214	40.2
	R: TCTTCTCTTCATCCGCTTC	19			
ZB-69	F: CCTACGCCAACTCACC	16	58	149	65.1
	R: ATGCGAACCTTCAACGTC	18			
ZB-73	F: TTGAGGACGACGAAGATG	18	56	201	51.4
	R: CTTCTTCCCCAGTCTCTC	18			
ZB-89	F: GATGTTGCGAATGATACAAG	20	54	100	46
	R: GACAATATTCAGGAGCAGGA	20			
ZB-97	F: AGTATTGTGGAGCTAACAT	19	54	191	38.2
	R: TGAGGCCATCTTTACACT	18			

**Figure 7:**
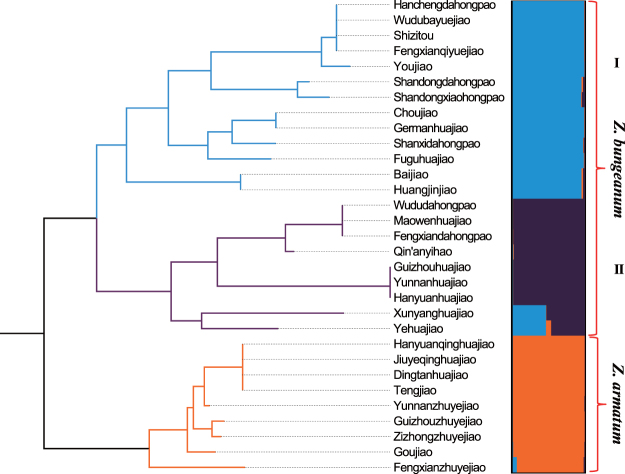
Cluster analysis of 31 cultivars and landraces of *Z*. *bungeanum* and *Z*. *armatum* based on 11 polymorphic SSR markers. (**A**) NJ-tree genetic relationships among *Zanthoxylum* cultivars and landraces. (**B**) STRUCTURE-based clusters resolved 31 cultivars and landraces into three groups (*K* = 3), *Z*. *bungeanum* I and II, and *Z*. *armatum*. Color groups correspond to NJ-tree clades.

Neighbor-joining tree and STRUCTURE analyses clearly grouped the 31 cultivars and landraces into three major clusters (Fig. 7). Cluster I (cyan) included 13 cultivars of *Z*. *bungeanum* mainly distributed north of the Qinling Mountains. Cluster II (purple) included nine *Z*. *bungeanum* cultivars mainly distributed south of the Qinling Mountains. These two clusters are commonly known as “Red huajiao” for their bright red pericarp. Cluster III (orange) consisted of nine cultivars of *Z*. *armatum*, commonly known as “Green huajiao” for their bright green pericarp.

## Discussion

Here we report the first transcriptome analysis of *Zanthoxylum*, an economic crop of great importance because of its flavor and industrial and medical applications^18^. The transcriptome sequence of *Z. bungeanum* provides a genomic resource for genetic study and breeding improvement of *Zanthoxylum*, and a reference for plant transcriptome-scale evolutionary analyses of the Rutaceae family.

Since *Z. bungeanum* has been bred for more than 4,000 years, a large number of elite cultivars have been widely dispersed, named, and released in China. Sequencing of the *Z. bungeanum* transcriptome provides genomic data for comprehensively assessing the heterozygosity of *Zanthoxylum* plants. Our transcriptome analysis indicated that *Z. bungeanum* might have a high degree of heterozygosity. Heterozygosity is a common feature in most plants and has important biological functions in crops. The heterozygosity of *Z*. *bungeanum*, estimated in the present study, is higher than that of sweet orange^17^, which suggests *Z*. *bungeanum* may have undergone an intensive inter- or intra-specific hybridization. Moreover, the high values of mean *H*_*o*_ and *H*_*e*_ also suggest a relatively high heterozygosity, while the negative value of *F*_*IS*_ also indicates a significant heterozygote excess in *Z*. *bungeanum*. Therefore, it is not feasible to consider whole-genome sequencing of *Zanthoxylum*. Fortunately, the advent of RNA-Seq is useful for collecting genetic information from *Z. bungeanum*.

GO functional classification may help us to understand the distribution of gene functions at the macro level, and to predict the physiological role of each unigene. KOG is a database in which orthologous gene products are classified^19^. In the present study, the annotated unigenes were classified into 25 KOG and 43 GO subterms or subcategories, suggesting that our transcriptome data for *Z. bungeanum* represented a wide diversity of transcripts^20,21^. Meanwhile, the results revealed that the assembled unigenes had diverse molecular functions and were involved in many metabolic pathways. Under the biological process category following GO classification, cellular process and metabolic process were prominent, indicating the occurrence of important cellular and metabolic activities in *Z*. *bungeanum*.

KEGG pathways are helpful to better understand biological function and gene interaction. Functional classification based on KEGG pathways indicated that numerous important metabolic pathways in *Z. bungeanum* were unknown and worth investigation. Obviously, “metabolic pathways” represented the largest category, suggesting *Z*. *bungeanum* invests in cell maintenance and defense. Moreover, unigenes associated with metabolism of cofactors and vitamins, terpenoids and polyketides, and biosynthesis of other secondary metabolites were identified in the present study, with evidence that numerous biologically active secondary metabolites have been isolated in *Zanthoxylum* species^18^. ‘Human diseases’ represented another dominant pathway and contained six different subcategories, indicating a complex composition of the *Zanthoxylum* genome, which makes it an important target for genomic study.

Phylogenetic analysis based on nuclear and chloroplast genes indicated that *Zanthoxylum* is relatively recent and estimated to have diverged from *Citrus* 36.5–37.7 MYA. Interestingly, the nuclear phylogeny showed that *Arabidopsis* was not linked to Malvidae with full bootstrap value support, whereas the chloroplast tree topology demonstrated that *Arabidopsis* was included in the Malvidae. This contradiction may be due to the difference in evolutionary pattern between or within nuclear and chloroplast genes, and the sensitivity of the topology to the maximum likelihood methods.

The WGD is predominant in most flowering plant species during evolution^22^, as gene duplication has provided a necessary source of material for the origin of evolutionary novelties. Due to functional redundancy, genes are rapidly silenced or lost from the duplicated genomes^23^. The detection method of plant WGDs is derived from genomic structure (*e.g*., syntenic analysis), phylogenetic tree construction, and evolutionary distances (*e.g*., synonymous mutation rate, *Ks*)^24,25^. To investigate the genome evolutionary history of *Zanthoxylum*, we estimated the WGD time in the *Zanthoxylum* genome by calculating the *Ks* age distribution using the formula T = *Ks*/2r. Here we chose r = 2.6 × 10^−9^ as the neutral substitution rate, because *Zanthoxylum* species are long-lived perennials and the generation time is negatively correlated with molecular evolutionary rates^26^. The synonymous mutation rates of several plants have been estimated using a small set of genes (2.6 × 10^−9^–1.5 × 10^−8^)^27^. It is likely that the slower rates may be more appropriate for *Zanthoxylum*. As for the transformation of the *Ks* values to absolute ages, low levels of *Ks* produce a more accurate age estimation than high *Ks* values because of possible substitution-saturation effects and molecular rate heterogeneity among lineages. In the present study, we deduced a recent WDG event in *Zanthoxylum* 14 MYA. We also calculated the *Ks* of each pair of three-copy and four-copy genes in *Zanthoxylum* and orange. Strikingly, they presented a similar but flatter curve than that of two-copy gene pairs, providing support for our confident estimation of *Ks* distribution and a more ancient duplication for multicopy genes.

In the present study, trinucleotide repeats were the most abundant type of SSR motifs in the transcriptome of *Z*. *bungeanum*, followed by the di-type motifs. This finding is similar to the results reported for pummelo^28^ and cotton^29^. Two hundred and six SSRs were present in the compound types, meaning they contained stretches of two or more different repeats. Among mononucleotide repeats, as in most plants, the A/T repeats were far more abundant than G/C repeats^30,31^. For the dinucleotide repeat category, the most abundant motif was AG/CT, which is similar to previous results in *Hemarthria compressa* “Yaan” and *H. altissima* “1110”^32^, *Ipomoea batatas* ^33^, *Brachypodium distachyon*, and *Oryza sativa* ^34^. Thirty-one motifs were found within the trinucleotide repeats; GAA/TTC and AAG/CTT were the most abundant, similar to results for colored calla lily^35^. These results support previous observations that dinucleotide AG/CT motifs and trinucleotide AAG/CTT motifs are the most abundant SSR motifs in dicots^36^.

Mining transcriptome data is an efficient way for SSR marker development and offers great flexibility in selecting markers for different applications at different resolutions^37^. Moreover, SSRs derived from a transcriptome database have advantages over other strategies, such as low cost and short time. In the present study, we verified and obtained 11 variety-specific EST-SSR markers (Table 2), which provides a simple, informative, reproducible, and suitable approach for evaluation of genetic relationships in *Zanthoxylum* species. The neighbor-joining tree and STRUCTURE analyses divided 31 *Zanthoxylum* cultivars and landraces into three clear clusters. The availability of the *Z. bungeanum* transcriptome and deep exploitation of *Zanthoxylum*-specific genes with known or unknown biochemical functions will facilitate a better understanding of agronomically and economically important traits, including yield, numb-taste components, oil, and stress resistance in subsequent research.

## Materials and Methods

### Plant material

*Z*. *bungeanum* cultivar “Fengxiandahongpao” with excellent pericarp was used in this study. At the sprouting stage, we collected 40 stem tips (1 cm) from five 7-year-old individual plants of *Z. bungeanum* from the Research Centre for Engineering and Technology of *Zanthoxylum*, State Forestry Administration, Northwest A&F University (Fengxian, Shaanxi Province, China). All stem tips were immediately frozen in liquid nitrogen and stored at −80 °C to preserve RNA for transcriptome sequencing. Currently, approximately 30 cultivars of *Z. bungeanum* and nine cultivars of *Z. armatum* are planted in China. A total of 31 cultivars and landraces, including *Z. bungeanum* (20 cultivars and two landraces) and *Z. armatum* (five cultivers and four landraces, Supplement Table S1), were collected from the main cultivated regions and used for estimating the SSR markers and cluster analysis.

### RNA isolation, integrity examination and RNA-Seq library preparation

Total RNA was extracted from each group using TRIzol Regent (Invitrogen, Life Technologies, Carlsbad, CA, USA) according to the manufacturer’s instructions. The extracted RNA was treated with a Total RNA Purification Kit (LC science, USA) for 30 min at 37 °C to remove any residual DNA. The quality and quantity of RNA were detected using a 2100 Bioanalyzer (Agilent Technologies, Santa Clara, CA, USA).

After total RNA extraction, the mRNA in each group was purified and enriched using oligo (dT) magnetic beads. Following purification, a fragmentation buffer and elevated temperatures were used to break the mRNA into fragments of 200–300 bp using divalent cations. Subsequently, the cleaved RNA fragments were employed for first-strand cDNA synthesis using reverse transcriptase and random primers, and then the second-strand cDNA was synthesized using DNA polymerase I and RNaseH (Invitrogen). Double stranded cDNAs then underwent an end repair process with T4 DNA polymerase and Klenow DNA polymerase. These repaired cDNA fragments were then subjected to 3ʹ- single adenylation and ligated with sequencing adapters. These products were purified and amplified via PCR to construct a cDNA library for transcriptome sequencing. Finally, the cDNA library products were sequenced on a paired-end flow cell using an Illumina HiSeq™ 2500 platform (Illumina Inc., San Diego, CA, USA).

### Assembly and functional annotation

The raw reads of cDNA libraries were cleaned by a stringent filtering process. All adapter fragments as well as ambiguous reads containing >5% unknown nucleotides and low-quality reads with more than 20% Q < 10 bases were removed to ensure the accuracy of *de novo* assembly and subsequent analyses. The clean reads were assembled using Trinity software^10^ with the parameters set at K-mer length of 25 and a similarity of 80%, with the other parameters set to their default values. The clean reads with a certain length of overlap were first joined to form longer fragments without N, known as contigs. Subsequently, the clean reads were mapped back to the contigs, and paired-end reads were used to calculate the distance and relation among these contigs. Trinity connected these contigs to obtain consensus sequences that could not be extended on either end, called transcripts. Finally, the transcripts were clustered into unigenes using TGI Clustering Tools^38^. Assembled sequences of less than 200 bp were discarded due to a low annotation rate.

All unigenes were subjected to BLASTx searches against the NCBI Nr database, the Swiss-Prot database, KOG, and KEGG with an *E*-value threshold of 10^−5^. According to the Nr annotation, the functional annotations of unigenes were obtained based on GO terms (GO annotation: http://www.geneontology.org) by employing Blast2GO^39^.

### Evolutionary analysis of CDS

Genescan^40^ was employed for gene prediction from unigenes. In total, 22,212 open reading frames were detected. The searching of single-copy homologous genes was performed by comparing the protein sequences of *Zanthoxylum*, sweet orange (*Citrus sinensis*), poplar (*Populus euphratica*), grape (*Vitis aestivalis*), strawberry (*Fragaria* × *ananassa*), soybean (*Glycine max*), *Arabidopsis*, cacao (*Theobroma cacao*), peach (*Prunus persica*), and castor bean (*Ricinus communis*), using all-to-all BLASTP analysis (*E*-value < 1E^−5^). Based on the species distribution of the top BLASTx hits against the Nr database, we selected sweet orange, poplar, grape, strawberry, soybean, *Arabidopsis*, cacao, peach, and castor bean for comparative phylogenetic analysis. Of these, *Zanthoxylum*, sweet orange, *Arabidopsis*, cacao, poplar, and castor bean belong to the Malvidae, a related phytogroup of Angiosperm Phylogeny Group classification. Rice (*Oryza sativa*) and sorghum (*Sorghum bicolor*) were used as out-groups. The OrthoMCL^41^ method was adopted to construct gene families for the 12 plant species. The translated putative amino acid sequences of the 34 shared single-copy genes were aligned in MAFFT^42^ and adjusted manually. Based on the concatenated sequences, phylogenetic analysis using the maximum-likelihood method was conducted in FastTree^43^ with the JTT model plus gamma-distributed rates and 1,000 bootstrap replications. For comparison, we also used peptide sequences of 68 genes from chloroplast genomes from the same plant species and performed maximum-likelihood analysis with the same parameters.

To detect the signature of genome duplications, an OrthoMCL clustering program was run on the protein CDS from the *Zanthoxylum* and *Citrus* genomes. One-to-one orthologous genes were then extracted from the clustering results. Subsequently, we set up a stringent criterion of at least 300 nucleotide alignments and 70% identity to reliably define two sequences as orthologous. Gene pairs with a BLAST identity of ≥99.0% were defined as identical sequences because near identical sequences occasionally are present in clustered transcript data due to sequencing errors and the fragmentary nature of transcripts. All identical gene pairs that contained the shorter sequence were discarded. The codon based alignment of the nucleotide sequence pairs was used to calculate the *Ks* by *KaKs*_Calculator^44^. The duplication time of the genome was estimated using the formula T = *Ks*/2r, where ‘r’ is the neutral substitution rate. A neutral substitution rate of 2.6 × 10^−9^ was used in this study^27^. The *Ks* of gene pairs of three-copy and four-copy genes families were also calculated.

### Variant analysis

Only reliable clean reads were considered for SNP calling. Indels were not considered because alternative splicing impedes reliable indel discovery^17^. SNPs were called using the SAMtools software package^45^. The assembled transcript sequences were used as templates to BLAST the clean reads.

### SSR locus detection and primer design

SSR locus screening was performed using the Perl script MIcroSAtellite (MISA, http://pgrc.ipk-gatersleben.de/misa/) based on the flanking sequences. SSR loci consist of repeated core sequences of two to six nucleotide motifs with minimum repeats of 6, 5, 4, 3 and 3, respectively. Mononucleotide repeats and complex SSRs were excluded from further analyses, because mononucleotide repeats may not be accurate due to sequencing errors and assembly mistakes, whereas complex SSRs show the least polymorphism. Primers could not be designed for those SSR loci whose flanking sequences were either too short or where the sequence did not meet appropriate criteria for primer design.

The SSR primer pairs were designed using the Oligo 7.0 program^46^ based on the following parameters: 1) primer length of 17–25 bp with 20 bp as the optimum; 2) PCR product size ranging from 100 to 500 bp; 3) GC content of 40–70% with an optimum of 50%; 4) melting temperature (T_m_) between 52 °C and 60 °C with 55 °C as the optimum annealing temperature, with a difference of no greater than 4 °C between the T_m_ values of the forward and reverse primers; 5) primer pairs devoid of secondary structure or consecutive tracts of a single nucleotide; 6) the designed primer sequence was limited to the middle region, with 30 bp being removed from the ends of the contig sequence^47^. In total, 100 primers for SSR markers were synthesized.

### DNA isolation, PCR amplification and SSR validation

The total genomic DNA was extracted from dried-leaf tissue of 31 cultivars and landraces of *Z*. *bungeanum* and *Z. armatum* (Supplement Table S1) using a modified CTAB protocol^48^. All SSR primer pairs were tested for amplification and polymorphism using the mixed DNA^49^ from five cultivars: ‘Fengxiandahongpao’, ‘Hanchengdahongpao’, ‘Huangjinjiao’, ‘Dingtanhuajiao’, and ‘Qinghuajiao’ (Supplement Table S1). In total, 100 pairs of primers were designed and validated by PCR and electrophoresis. Amplification by PCR was performed using an A200 Gradient Thermal cycler (LongGene, China), with reactions consisting of 10 µL of 2× *Taq* MasterMix (CWBIO Biotechnology, Beijing, China); 0.5 µL of each primer (100 µmol/L), 8 µL of double distilled water, and 1.0 µL of genomic DNA in a final reaction volume of 20 µL. The PCR conditions were: initial denaturation at 94 °C for 4 min, followed by 30 cycles of 1 min at 94 °C, 50 s at 52 to 60 °C (depending on the T_m_ of the primer pair used), and 1 min at 72 °C; then a final extension for 7 min at 72 °C. The resulting PCR products were subjected to electrophoresis on 12% non-denaturing polyacrylamide gels at 250 V for 2–2.5 h and stained using a silver-staining protocol^50^. Cluster analysis was conducted and displayed using the neighbor-joining algorithm and STRUCTURE analysis as implemented in MEGA ver. 6.0^51^ and STRUCTURE ver. 2.3.4^52^. The genetic diversity parameters (*N*_*a*_, *N*_*e*_, *H*_*o*_, *H*_*e*_, *I*, and *F*_*IS*_) of selected SSR primer pairs were calculated using the POPGENE software^53^.

### Data Availability

Transcriptome datasets supporting the conclusions of this article are available in the NCBI SRA repository under the accession number SRR5114371. The full dataset is also available from Shijing Feng upon request (shijing0554010112@163.com).

## Electronic supplementary material

Supplementary Information

## References

[1] Huang C (1997). Reipublicae popularis sinicae. In: Delectis Florae Reipublicae Popularis Sinicae agendae academiae sinicae edita. Beijing: Science Press.

[2] Kubitzki, K., Kallunki, J. A., Duretto, M. & Wilson, P. G. Rutaceae. In: Kubitzki, K. (Ed.), The Families and Genera of Vascular Plants, Flowering Plants Eudicots:Sapindales, Cucurbitales, Myrtaceae. *Berlin: Springer Verlag*, pp. 276–356 (2011).

[3] Yang X (2008). Aroma constituents and alkylamides of red and green huajiao (*Zanthoxylum bungeanum* and *Zanthoxylum schinifolium)*. Journal of Agricultural & Food Chemistry.

[4] Ahsan M (2014). Cytotoxic dimeric quinolone–terpene alkaloids from the root bark of *Zanthoxylum rhetsa*. Phytochemistry.

[5] Zhang Y, Wang D, Yang L, Zhou D, Zhang J (2014). Purification and characterization of flavonoids from the leaves of *Zanthoxylum bungeanum* and correlation between their structure and antioxidant activity. Plos One.

[6] Xia L, You J, Li G, Sun Z, Suo Y (2011). Compositional and antioxidant activity analysis of *Zanthoxylum bungeanum* seed oil obtained by supercritical CO_2_ fluid extraction. Journal of the American Oil Chemists’ Society.

[7] Artaria C, Maramaldi G, Bonfigli A, Rigano L, Appendino G (2011). Lifting properties of the alkamide fraction from the fruit husks of *Zanthoxylum bungeanum*. International Journal of Cosmetic Science.

[8] Tezuka Y (2001). Screening of Chinese herbal drug extracts for inhibitory activity on nitric oxide production and identification of an active compound of *Zanthoxylum bungeanum*. Journal of Ethnopharmacology.

[9] Liu Y, Wang F, Qin N (1987). Apomixis in *Zanthoxylum bungeanum* and *Z. simulans*. Journal of Genetics & Genomics.

[10] Grabherr MG (2011). Full-length transcriptome assembly from RNA-Seq data without a reference genome. Nature Biotechnology.

[11] Wang M, Zhang X, Liu JH (2015). Deep sequencing-based characterization of transcriptome of trifoliate orange (*Poncirus trifoliata* (L.) Raf.) in response to cold stress. BMC Genomics.

[12] Ma Y, Li Q, Hu G, Qin Y (2017). Comparative transcriptional survey between self-incompatibility and self-compatibility in *Citrus* reticulata Blanco. Gene.

[13] Liu XY, Li J, Liu MM, Yao Q, Chen JZ (2017). Transcriptome profiling to understand the effect of *Citrus* rootstocks on the growth of ‘Shatangju’ Mandarin. Plos One.

[14] Lu X (2016). Comparative transcriptome analysis reveals a global insight into molecular processes regulating citrate accumulation in sweet orange (*Citrus sinensis*). Physiologia Plantarum.

[15] Franco MC, Schimpl FC, Mazzafera P (2017). Reference genes for quantitative RT-PCR of the pilocarpine producer *Pilocarpus* microphyllus and two other *Pilocarpus* species. Theoretical & Experimental Plant Physiology.

[16] Cheng J (2015). Biomass accumulation and carbon sequestration in an age-sequence of *Zanthoxylum bungeanum* plantations under the Grain for Green Program in karst regions, Guizhou province. Agricultural & Forest Meteorology.

[17] Xu Q (2013). The draft genome of sweet orange (*Citrus sinensis*). Nature Genetics.

[18] PatiñO, L. O. J., Prieto, R. J. A. L. & Cuca, S. L. E. Bioactive compounds in phytomedicine: *Zanthoxylum* genus as potential source of bioactive compounds, pp. 185–218. In: Rasooli I. (ed.), *InTech*, *Rijeka Croatia* (2008).

[19] Kumar S, Shah N, Garg V, Bhatia S (2014). Large scale in-silico identification and characterization of simple sequence repeats (SSRs) from de novo assembled transcriptome of *Catharanthus roseus* (L.) G. Don. Plant Cell Reports.

[20] Zhou T (2016). Transcriptome sequencing and development of genic SSR markers of an endangered Chinese endemic genus *Dipteronia* Oliver (Aceraceae). Molecules.

[21] Yang Y (2015). Phenotype and transcriptome analysis reveals chloroplast development and pigment biosynthesis together influenced the leaf color formation in mutants of *Anthurium andraeanum* ‘Sonate’. Frontiers in Plant Science.

[22] Jiao Y (2011). Ancestral polyploidy in seed plants and angiosperms. Nature.

[23] Shi T, Huang HW, Barker MS, Heslopharrison JSP (2010). Ancient genome duplications during the evolution of kiwifruit (*Actinidia*) and related Ericales. Annals of Botany.

[24] Peer YVD, Fawcett JA, Proost S, Sterck L, Vandepoele K (2009). The flowering world: a tale of duplications. Trends in Plant Science.

[25] Tang H (2008). Synteny and collinearity in plant genomes. Science.

[26] Gaut, B. S. Molecular clocks and nucleotide substitution rates in higher plants. pp. 93–120 in Hecht, M. K. ed. *Evolutionary biology*. Plenum Press, New York (1998).

[27] Senchina DS (2003). Rate variation among nuclear genes and the age of polyploidy in *Gossypium*. Molecular Biology & Evolution.

[28] Mei L (2015). *De Novo* transcriptome assembly of pummelo and molecular marker development. Plos One.

[29] Zhang X (2014). Characterization of the global transcriptome for cotton (*Gossypium hirsutum* L.) anther and development of SSR marker. Gene.

[30] Yue H (2016). *De novo* assembly and characterization of the transcriptome of broomcorn millet (*Panicum miliaceum* L.) for gene discovery and marker development. Frontiers in Plant Science.

[31] Gao Z (2013). Rapid microsatellite development for tree peony and its implications. BMC Genomics.

[32] Huang X (2016). *De novo* transcriptome analysis and molecular marker development of two *Hemarthria* species. Frontiers in Plant Science.

[33] Wang Z (2010). *De novo* assembly and characterization of root transcriptome using Illumina paired-end sequencing and development of cSSR markers in sweet potato (*Ipomoea batatas*). BMC Genomics.

[34] Asp T, Frei UK, Didion T, Nielsen KK, Lübberstedt T (2007). Frequency, type, and distribution of EST-SSRs from three genotypes of *Lolium perenne*, and their conservation across orthologous sequences of *Festuca arundinacea*, *Brachypodium distachyon*, and *Oryza sativa*. BMC Plant Biology.

[35] Wei Z (2016). Transcriptome analysis of colored calla lily (*Zantedeschia rehmannii* Engl.) by Illumina sequencing: *de novo* assembly, annotation and EST-SSR marker development. Peer j.

[36] Chen LY (2015). Characterization of transcriptome and development of novel EST-SSR makers based on next-generation sequencing technology in *Neolitsea sericea* (Lauraceae) endemic to East Asian land-bridge islands. Molecular Breeding.

[37] Jin YQ (2016). Genetic evaluation of the breeding population of a valuable reforestation conifer *Platycladus orientalis* (Cupressaceae). Scientific Reports.

[38] Pertea G (2003). TIGR Gene Indices clustering tools (TGICL): a software system for fast clustering of large EST datasets. Bioinformatics.

[39] Conesa A (2005). Blast2GO: a universal tool for annotation, visualization and analysis in functional genomics research. Bioinformatics.

[40] Tiwari S, Ramachandran S, Bhattacharya A, Ramaswamy R (1997). Prediction of probable genes by Fourier analysis of genomic sequences. Bioinformatics.

[41] Li L, Stoeckert CJ, Roos DS (2003). OrthoMCL: Identification of ortholog groups for eukaryotic genomes. Genome Research.

[42] Katoh K, Standley DM (2013). MAFFT multiple sequence alignment software version 7: improvements in performance and usability. Molecular Biology & Evolution.

[43] Price MN, Dehal PS, Arkin AP (2010). FastTree 2-approximately maximum-likelihood trees for large alignments. Plos One.

[44] Wang D, Zhang Y, Zhang Z, Jiang Z, Yu J (2010). KaKs_Calculator 2.0: A toolkit incorporating Gamma-Series methods and sliding window strategies. Genomics, Proteomics & Bioinformatics.

[45] Li H (2009). The Sequence alignment/map format and SAMtools. Bioinformatics.

[46] Rychlik W (2007). OLIGO 7 primer analysis software. Methods in Molecular Biology.

[47] Chen W, Liu YX, Jiang GF (2014). *De novo* assembly and characterization of the testis transcriptome and development of EST-SSR markers in the cockroach *Periplaneta americana*. Scientific Reports.

[48] Porebski S, Bailey LG, Baum BR (1997). Modification of a CTAB DNA extraction protocol for plants containing high polysaccharide and polyphenol components. Plant Molecular Biology Reporter.

[49] Liu G (2012). Studies on quick screening of EST-SSR primers based on genomic DNA mixing pool. Journal of Central South University of Forestry & Technology.

[50] Li Y, Wang SH, Li ZQ, Jin CF, Liu MH (2014). Genetic diversity and relationships among Chinese *Eucommia ulmoides* cultivars revealed by sequence-related amplified polymorphism, amplified fragment length polymorphism, and inter-simple sequence repeat markers. Genetics & Molecular Research.

[51] Tamura K, Stecher G, Peterson D, Filipski A, Kumar S (2013). MEGA6: Molecular Evolutionary Genetics Analysis version 6.0. Systematic Biology.

[52] Falush D, Stephens M, Pritchard JK (2007). Inference of population structure using multilocus genotype data: dominant markers and null alleles. Molecular Ecology Notes.

[53] Yeh, F. C., Yang, R. C. & Boyle, T. Population genetic analysis (POPGENE 1.31). A Joint Project of University of Alberta and Center for International Forestry Research, Alberta, Canada (1999).

