# Supplementary material for: De novo transcriptome assembly of Zanthoxylum bungeanum using Illumina sequencing for evolutionary analysis and simple sequence repeat marker development
Source: Sci Rep. 2017 Dec 1;7:16754. doi: 10.1038/s41598-017-15911-7 (PMC5711952; doi:10.1038/s41598-017-15911-7)
Supplement: Supplementary file 1 — Supplementary Information [file 41598_2017_15911_MOESM1_ESM.pdf]

De novo transcriptome assembly of *Zanthoxylum bungeanum* using Illumina sequencing for evolutionary analysis and simple sequence repeat marker development

Shijing Feng[1], Lili Zhao[1], Zhenshan Liu[2], Yulin Liu[1], Tuxi Yang[1] & Anzhi Wei[1]

[1]College of Forestry, Northwest A&F University, Yangling, Shaanxi 712100, China. [2]College of Life Science, Northwest A&F University, Yangling, Shaanxi 712100, China. Correspondence and requests for materials should be addressed to A.W. (email: weianzhi@126.com). [†]Co-first author: this author contributed equally to this paper

Supplement Table S1. The 31 cultivars of *Zanthoxylum bungeanum* and *Z. armatum* used in the present study.

Code	Cultivar	Species	Location
1	Fengxianqiyuejiao	*Z. bungeanum*	Fengxian, Shaanxi
2	Fengxiandahongpao	*Z. bungeanum*	
3	Fengxianzhuyejiao	*Z. armatum*	
4	Yehuajiao	*Z. bungeanum*	
5	Hanchengdahongpao	*Z. bungeanum*	Hancheng, Shaanxi
6	Shizitou	*Z. bungeanum*	
7	Choujiao	*Z. bungeanum*	
8	Fuguhuajiao	*Z. bungeanum*	Fugu, Shaanxi
9	Xunyanghuajiao	*Z. bungeanum*	Xunyang, Shaanxi
10	Goujiao	*Z. armatum*	
11	Wududahongpao	*Z. bungeanum*	Wudu, Gansu
12	Wudubayuejiao	*Z. bungeanum*	
13	Baijiao	*Z. bungeanum*	Qin'an, Gansu
14	Youjiao	*Z. bungeanum*	
15	Qin'anyihao	*Z. bungeanum*	
16	Hanyuanhuajiao	*Z. bungeanum*	Hanyuan, Sichuan
17	Maowenhuajiao	*Z. bungeanum*	
18	Hanyuanqinghuajiao	*Z. armatum*	
19	Zizhongzhuyejiao	*Z. armatum*	Zizhong, Sichuan
20	Tengjiao	*Z. armatum*	
21	Guizhouhuajiao	*Z. bungeanum*	Liupanshui, Guizhou
22	Guizhouzhuyejiao	*Z. armatum*	
23	Dingtanhuajiao	*Z. armatum*	Guanling, Guizhou
24	Yunnanhuajiao	*Z. bungeanum*	Kunming, Yunnan
25	Jiuyeqinghuajiao	*Z. armatum*	
26	Yunnanzhuyejiao	*Z. armatum*	
27	Shandongdahongpao	*Z. bungeanum*	Laiwu, Shandong
28	Shandongxiaohongpao	*Z. bungeanum*	
29	Huangjinjiao	*Z. bungeanum*	Wutai, Xinzhou
30	Shanxidahongpao	*Z. bungeanum*	
31	Germanhuajiao	*Z. bungeanum*	Germa

Supplement Table S2. The genetic diversity parameters of 11 SSR primer pairs.

	Primers	N_a	N_e	I	H_o	H_e	F_{IS}
Z. bungeanum	ZB2	2.000	1.658	0.586	0.546	0.406	-0.485
	ZB4	3.000	2.390	0.978	0.864	0.595	-0.375
	ZB11	5.000	4.246	1.523	1.000	0.782	-0.375
	ZB28	6.000	3.796	1.524	0.773	0.754	-0.308
	ZB30	6.000	4.840	1.641	1.000	0.812	-0.281
	ZB32	6.000	2.830	1.266	0.409	0.662	-0.260
	ZB43	7.000	3.270	1.514	0.955	0.710	-0.202
	ZB69	4.000	2.085	0.939	0.667	0.533	-0.199
	ZB73	4.000	1.972	0.937	0.591	0.504	-0.049
	ZB89	4.000	3.133	1.213	0.818	0.697	0.156
	ZB97	4.000	3.753	1.350	0.619	0.752	0.367
	Mean	4.636	3.088	1.225	0.749	0.655	-0.183
Z. armatum	ZB2	1.000	1.000	0.000	0.000	0.000	NA
	ZB4	2.000	1.385	0.451	0.111	0.294	0.600
	ZB11	4.000	1.421	0.634	0.222	0.314	0.250
	ZB28	3.000	1.742	0.730	0.444	0.451	-0.044
	ZB30	2.000	1.670	0.591	0.556	0.425	-0.385
	ZB32	1.000	1.000	0.000	0.000	0.000	NA
	ZB43	1.000	1.000	0.000	0.000	0.000	NA
	ZB69	1.000	1.000	0.000	0.000	0.000	NA
	ZB73	2.000	1.528	0.530	0.444	0.366	-0.286
	ZB89	2.000	1.976	0.687	0.889	0.523	-0.800
	ZB97	3.000	2.455	0.965	0.889	0.628	-0.500
	Mean	2.000	1.471	0.417	0.323	0.273	0.027

Note: N_a=Observed number of alleles, N_e=Effective number of alleles, I=Shannon's Information index, H_o=Observed heterozygosity, H_e=Expected heterozygosity, and F_{IS}=Fixation index

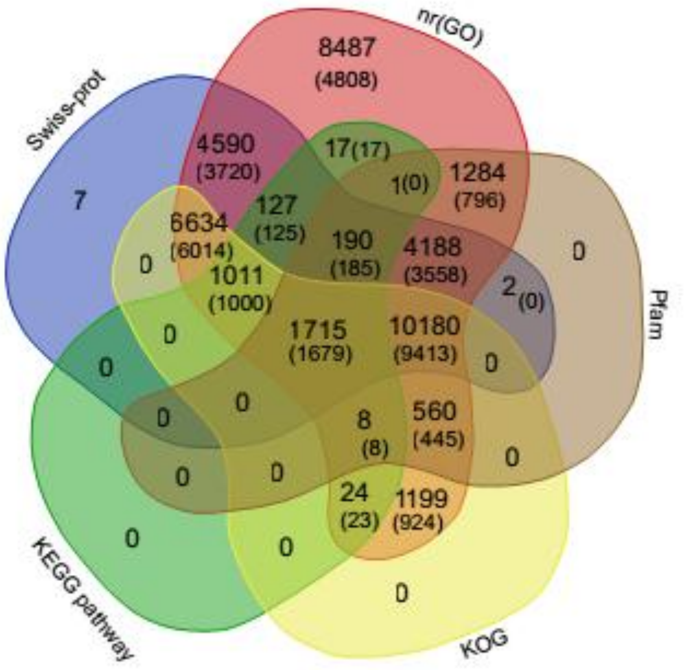

Supplement Figure S1. The number of *Z. bungeanum* unigenes annotated by BLASTX with an *E*-value threshold of 10^{-5} against protein database. The numbers in the circles indicate the number of unigenes annotated by single and multiple databases.

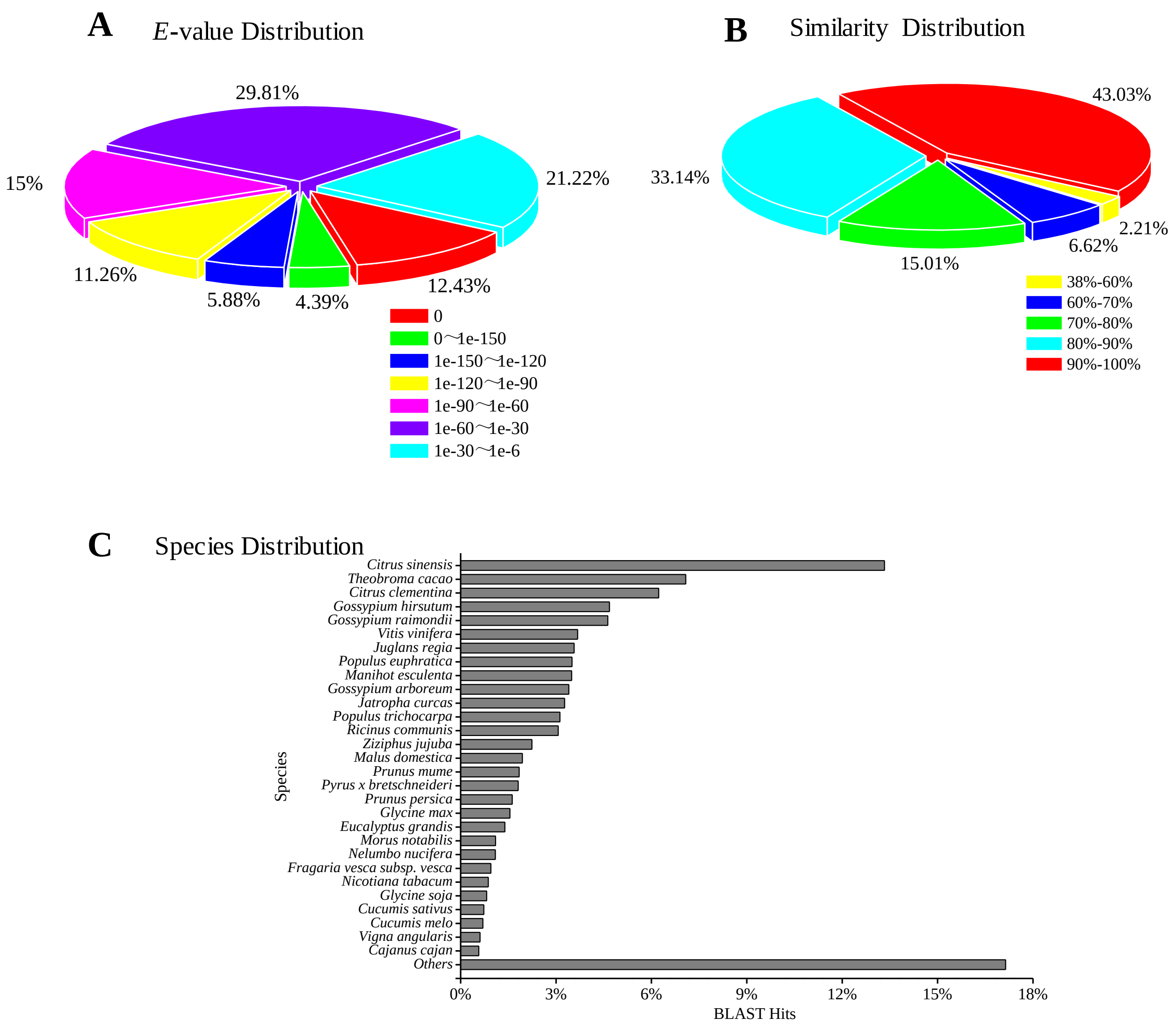

Supplement Figure S2. Characteristics of similarity of *Z. bungeanum* all-unigene against Nr database. (A) *E*-value distribution of all-unigene. (B) Similarity distribution of all-unigene. (C) Species distribution of all-unigene.

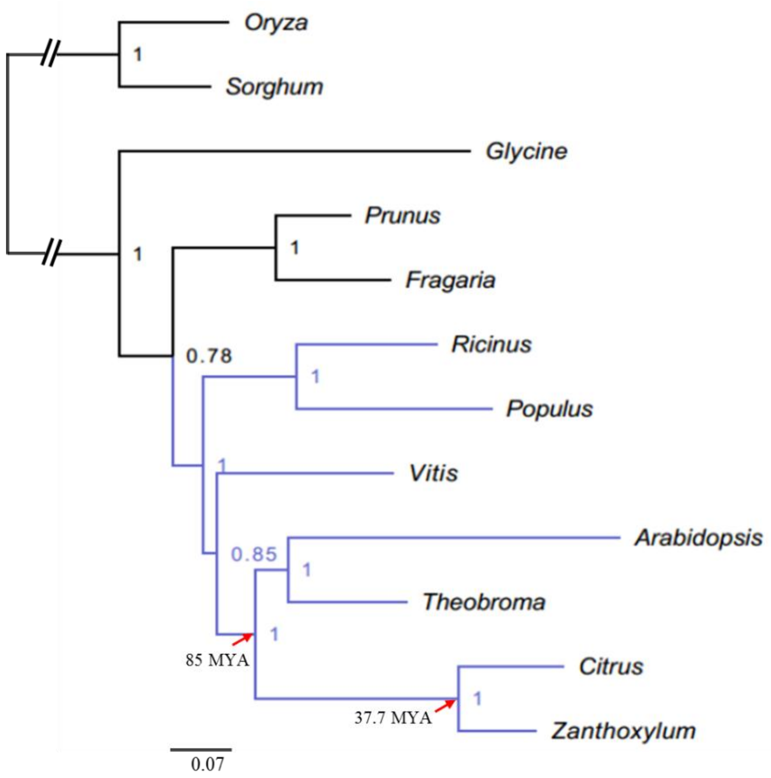

Supplement Figure S3. *Zanthoxylum* phylogeny on the basis of 68 chloroplast genes shared in *Zanthoxylum*, sweet orange (*Citrus sinensis*), poplar (*Populus euphratica*), grape (*Vitis aestivalis*), strawberry (*Fragaria × ananassa*), soybean (*Glycine max*), *Arabidopsis*, cacao (*Theobroma cacao*), peach (*Prunus persica*), castor bean (*Ricinus communis*). Rice (*Oryza sativa*) and sorghum (*Sorghum bicolor*) serve as out-groups. MYA, million years ago.

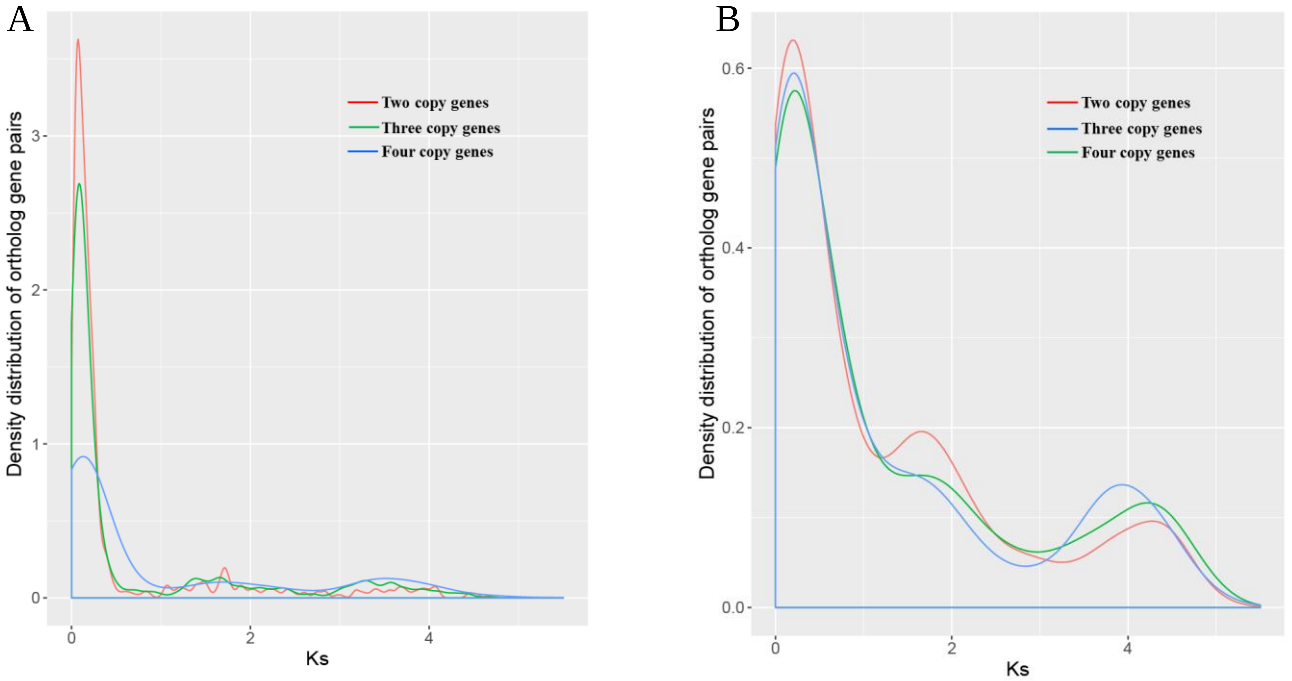

Supplement Figure S4. Distribution of synonymous substitution rates (*K*s) for gene pairs of multi-copy homologous genes of (A) *Zanthoxylum* and (B) *Citrus*.

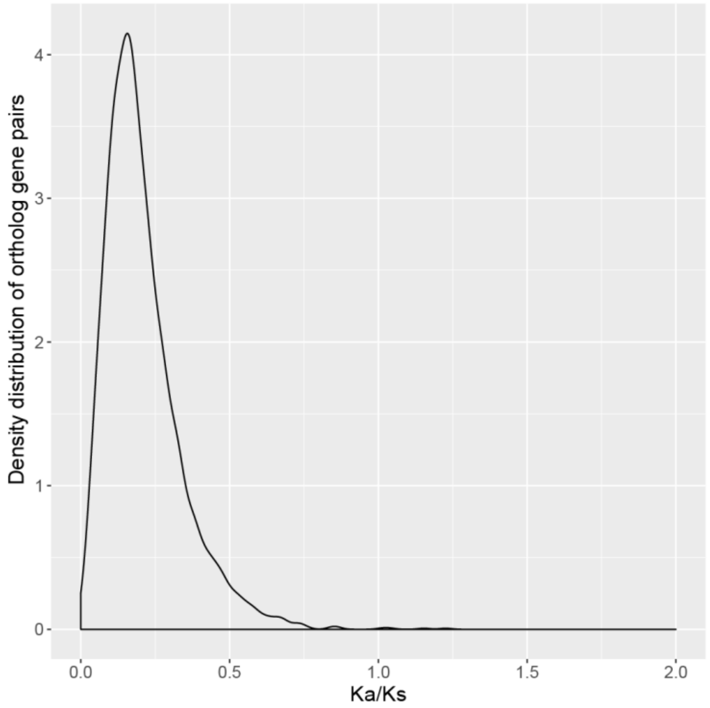

Supplement Figure S5. Ka/Ks distribution of *Zanthoyxlum* coding sequences by referring to the *Citrus* genome.

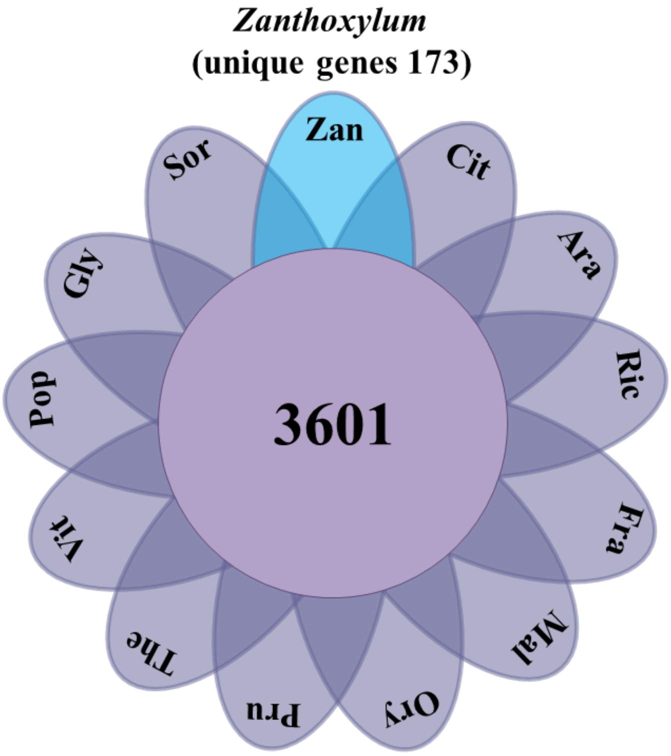

Supplement Figure S6. *Zanthoxylum*-specific genes identified by comparative analyses of *Zanthoxylum* protein coding sequences with 12 other plant genomes. Zan-*Zanthoxylum*, Cit-*Citrus*, Ara-*Arabidopsis*, Ric-*Ricinus*, Fra-*Fragaria*, Mal-*Malus*, Ory-*Oryza*, Pru-*Prunus*, The-*Theobroma*, Vit-*Vitis*, Pop-*Populus*, Gly-*Glycine*, and Sor-*Sorghum*.
